# Current attitudes and preconceptions on newborn genetic screening in the Chinese reproductive-aged population

**DOI:** 10.1186/s13023-022-02474-8

**Published:** 2022-08-26

**Authors:** Xin Wang, Xian-Wei Guan, Yan-Yun Wang, Zhi-Lei Zhang, Ya-Hong Li, Pei-Ying Yang, Yun Sun, Tao Jiang

**Affiliations:** Genetic Medicine Center, Women’s Hospital of Nanjing Medical University, Nanjing Maternity and Child Health Care Hospital, 123 Tianfei Lane, Mochou Road, Qinhuai District, Nanjing, 210004 Jiangsu Province China

**Keywords:** Newborn screening, Genetic screening, Inherited metabolic disorder, Rare disease

## Abstract

**Purpose:**

Newborn screening (NBS) applications are limited as they can only cover a few genetic diseases and may have false positive or false negative rates. A new detection program called newborn genetic screening (NBGS) has been designed to address the potential defects of NBS. This study aimed to investigate the perceptions, acceptance, and expectations of childbearing people related to NBGS to provide the basis for the targeted improvement in the NBGS program carried out in Hospitals.

**Methods:**

A questionnaire with 20 items was designed on www.wjx.cn. Individuals who came to the Nanjing maternity and child health care Hospital for consultation from June 2021 to August 2021 participated in the survey. The data of the study was arranged properly and analyzed after the investigation.

**Results:**

A total of 1141 valid questionnaires were collected in the survey, in which the average age of the participants was 31 (± 4) years, and a 1:4 ratio of males to females. Additionally, 65.12% of the participants possessed a bachelor's degree or above qualification. Overall, 50.57% of participants had an annual household income of 100,000–250,000 RMB, while about 86.68% of the participants supported the development of NBGS. The participation cost to pay for NBGS depended on the family incomes; about 59.42% of them were willing to pay a participation fee of 1000–2000 RMB.

**Conclusion:**

Our research provisionally demonstrated that the residents generally supported the use of NBGS, especially those with higher educational degrees, but the understanding of the genetic diseases and NBGS among the low-educated population still needs to be strengthened.

**Supplementary Information:**

The online version contains supplementary material available at 10.1186/s13023-022-02474-8.

## Introduction

The traditional newborn screening (NBS) program has a developmental history of more than 50 years [1]. It is a successful public health program that functions to find severe disorders early by detecting various biochemical indicators from heel blood samples in newborns [2]. Early detection, diagnosis, and intervention of severe disorders can help prevent deaths and improve children's quality of life with lethal newborn diseases [3].

Currently, the most commonly used method is the application of tandem mass spectrometry (MS–MS), which has a significant advantage in cost-effectiveness and high throughput. Unlike other previously used methods, it can effectively detect more than 40 inherited metabolic diseases (IMD) [4–7]. IMD of genetic diseases specifically refers to a type of genetic disease with various defects in the metabolic function, most of which are single-gene genetic diseases. These include macromolecular metabolic disorders such as lysosomal storage and mitochondrial diseases. It also includes small-molecule metabolic disorders involving amino acids, organic acids, and fatty acids [8]. Nonetheless, due to several drawbacks, such as false-positive rates and a restricted number of diseases associated with existing detection methods [9, 10], the IMD of newborns that can be detected is highly limited.

With the transformation of society, which includes advances in medical research and economic growth, emphasis has been given to prenatal and postnatal care services, and the demand for congenital disability prevention networks has increased. There is an immediate need to develop a comprehensive NBS program capable of detecting a more significant number of diseases with high accuracy [9]. It is also intended to promote early screening, diagnosis and treatment and improve the newborn population's quality of life. Next-generation sequencing (NGS) technology is used in newborn genetic screening (NBGS), which includes disease-targeted gene package panel sequencing, whole-exome sequencing, and whole-genome sequencing [11]. The NBGS can functionally detect the pathogenic genes of different diseases. This has greatly boosted the detection rate and has played a key role in excluding screening in some diseases based on biochemical testing. NBGS has various restrictions that may impede its implementation, including difficulties in interpreting variant sites, the privacy of genetic information, the higher cost, the possibility of missing some diseases that require biochemical identification, and the risk of overmedication caused by the rise in diseases [11]. The BabySeq exome-sequencing project (Boston, USA), the NC NEXUS exome-sequencing project (North Carolina, USA), the NBSeq project (California, USA), and the NESTS genetic screening project (Beijing, China) have been reported [12–17]. The findings of these programs have demonstrated that genetic screening can detect a wide variety of diseases and specific variants that NBS cannot detect and affirm the actual value of genetic screening. In addition, a detailed analysis was carried out on the inclusion conditions of the gene screening diseases and the interpretation criteria of the results [18, 19], which has provided a sound basis for further exploration of the clinical applications of NBGS.

It is crucial to determine if the NBGS program can be successfully implemented in China based on the acceptability of the relevant clinical population. Population support is critical for the rapid development of the project, but there are still no relevant reports published in China yet. Therefore, based on the existing genetic screening foundation, this study anticipated surveying the clinically relevant populations. Following that, through analysis of the Chinese population's cognition and acceptance of the NBGS projects, the clinical feasibility of the NBGS will be systematically evaluated to promote the clinical development of this technology.

## Materials and methods

### Questionnaire design

A questionnaire on the NBGS was designed with 20 different items. The questionnaire included the various basic information such as gender, age, education, family income of the participants, their understanding, views on the NBS program and genetic diseases, and their willingness and expectations of including an additional genetic screening in the NBS program. The Research Ethics Committee of the Women's Hospital of Nanjing Medical University (2021KY-071) approved the present study.

### Study population

After obtaining the participant's consent, we performed a questionnaire survey on several genetic screening-related problems for Nanjing women of reproductive ages who visited the Nanjing Maternity and Child Health Care Hospital's genetic therapy clinic. A total of 1141 valid questionnaires were acquired after excluding the non-Nanjing population.

### Statistical analyses

The data is expressed as the median ± standard deviation (± SD). A Chi-square test was used for the statistical comparisons. A *P*-value of < 0.05 was considered statistically significant (*P < 0.05; **P < 0.01; ***P < 0.001).

## Results

### Demographic information of the participants

**Fig. 1:**
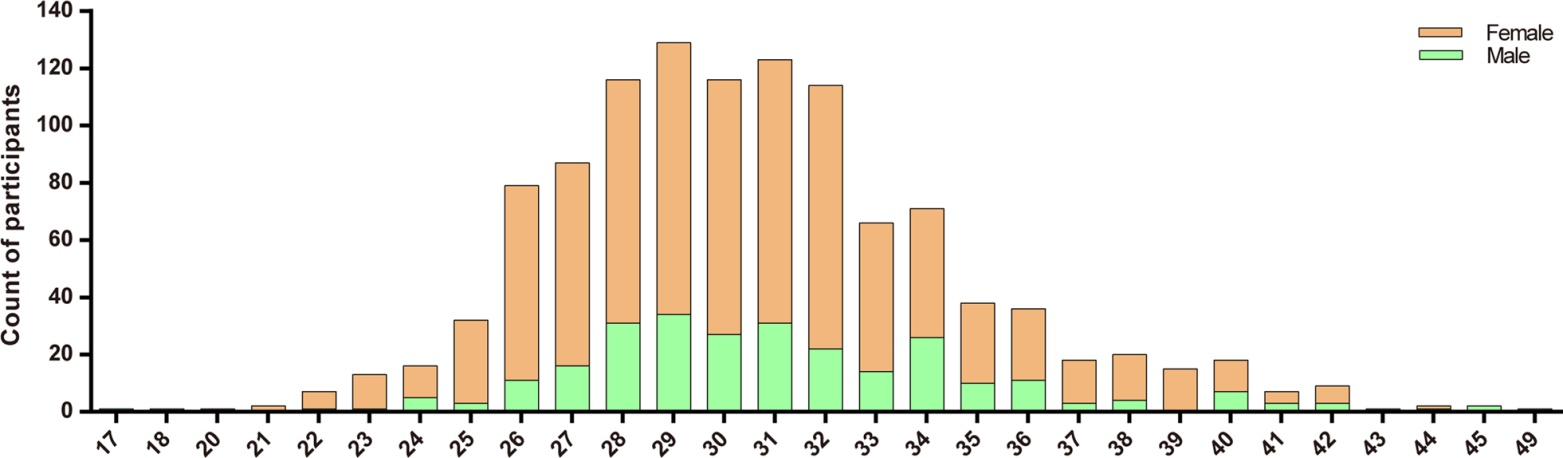
The distribution of participants of different ages

1141 individuals who intended to have a baby or a second child were given a questionnaire (Additional file 1: Table S1) to assess the population's perspectives on the NBGS. The data indicated that most participants who came for consultation were approximately 30 years old. The average age of the participants was 31 (± 4) years, with a male to female ratio of 1:4 (Fig. 1). About 99.74% of the participants did not have any family history of the genetic diseases. Among all the study participants, 65.12% possessed a bachelor's degree or above qualification. While half of the participants had an annual household income of around 100,000–250,000 RMB (50.57%) (Table 1).

**Table 1:** Characteristics of participants and gender

	Total (n = 1141, 100%)	Male (n = 271, 23.75%)	Female (n = 870, 76.25%)	Value	P
Age (years old)					
≤ 24	41 (3.59%)	9 (3.32%)	32 (3.68%)	2.942	P > 0.05
25–29	443 (38.83%)	95 (35.06%)	348 (40%)		
30–34	490 (42.94%)	121 (44.65%)	369 (42.41%)		
≥ 35	167 (14.64%)	46 (16.97%)	121 (13.91%)		
Have you had family history of genetic disease?					
Yes	3 (0.26%)	0 (0.00%)	3 (0.34%)	0.937	P > 0.05
No	1138 (99.74%)	271 (100%)	867 (99.66%)		
Educational background					
Below high school	53 (4.64%)	12 (4.43%)	41 (4.71%)	0.509	P > 0.05
High school/technical secondary school	103 (9.03%)	22 (8.12%)	81 (9.31%)		
Junior college/ Vocational College	242 (21.21%)	60 (22.14%)	182 (20.92%)		
Undergraduate or above	743 (65.12%)	177 (65.31%)	566 (65.06%)		
Family income					
< 100 thousand RMB	187 (16.39%)	49 (18.08%)	138 (15.86%)	0.890	P > 0.05
100–250 thousand RMB	577 (50.57%)	134 (49.45%)	443 (50.92%)		
260–400 thousand RMB	255 (22.35%)	61 (22.51%)	194 (22.30%)		
> 400 thousand RMB	122 (10.69%)	27 (9.96%)	95 (10.92%)		
Nation					
Han nationality	1117 (97.90%)	267 (98.52%)	850 (97.70%)	0.679	P > 0.05
Others	24 (2.10%)	4 (1.48%)	20 (2.30%)		
Had any abnormalities in you/your spouse's fetus during pregnancy?					
Yes	34 (2.98%)	10 (3.69%)	24 (2.76%)	0.620	P > 0.05
No	1107 (97.02%)	261 (96.31%)	846 (97.24%)		

### Awareness of NBS

Most families had a relatively sufficient understanding of the genetic diseases and were aware of the aims of the NBS project (Table 2). When the NBS results were abnormal, most participants could make the correct choice regarding the time, willing to actively cooperate with further testing, which indicated a positive attitude towards NBS (99.12%).

**Table 2:** Compliance of participants with NBS

	Is it necessary to undergo genetic screening?			Value	P
	Total (n = 1141, 100%)	Yes (n = 989, 86.68%)	No/I don’t know (n = 152, 13.32%)		
Do you think if both couples do not have genetic diseases, their child will not have a genetic disease?					
Yes	117 (10.25%)	93 (9.40%)	24 (15.79%)		
No	738 (64.68%)	673 (68.05%)	65 (42.76%)	37.038	P < 0.05
I don't know	286 (25.07%)	223 (23.56%)	63 (41.45%)		
If the NBS results are negative, does it mean that the baby will not have an IMD?					
Yes	127 (11.13%)	108 (10.92%)	19 (12.50%)		
No	647 (56.70%)	594 (60.06%)	53 (34.87%)	37.879	P < 0.05
I don't know	367 (32.17%)	287 (29.02%)	80 (52.63%)		
Do you know what it means to be suspiciously positive for NBS?					
It means that there have abnormal indicators and needs to be further re-examined	1052 (92.20%)	935 (94.54%)	117 (76.97%)	67.085	P < 0.05
It means that a disease has been diagnosed	15 (1.31%)	13 (1.31%)	2 (1.31%)		
I don't know, it’s probably meaningless	74 (6.49%)	41 (4.15%)	33 (21.71%)		
If your child's neonatal disease screening is suspiciously positive, how would you treat it?					
No treatment until the child has symptoms	5 (0.44%)	3 (0.30%)	2 (1.31%)		
Search information on the Internet and treat by oneself	5 (0.44%)	4 (0.40%)	1 (0.66%)	3.296	P > 0.05
Go to the appointed hospital or department immediately	1131 (99.12%)	982 (99.30%)	149 (98.03%)		

*IMD* inherited metabolic diseases, *NBS* newborn screening

### Expectations for NBS

Additionally, regarding the further development of NBS, most of the participants gave a positive answer (81.51%) on whether there was a need to further increase the number of NBS diseases. The people who supported the NBGS were considered the prominent supporters (87.06%), and about half of the remaining people (44.08%) wanted to seek the doctor's opinion first.

In terms of increasing the number of diseases, most participants (71.96%) assumed that the more the better. Approximately 22.52% of the population held a more conservative attitude and believed that 10–50 different types of diseases could be added based on the originally selected diseases. But, in the non-supportive NBGS population, 21.71% of the population believed that there was no need to increase the number of diseases. Moreover, for the detection rate of new diseases added to NBS, the participants (54.78%) considered that a relatively satisfactory NBGS program should have a total detection rate of at least 95%. For the diseases with a very low incidence, the detection rate should reach at least 80% (51.36%). Among them, the people who supported the NBGS generally had higher requirements for the detection rate than the rest of the population. The participants were also willing to bear the costs arising from the increase in diseases, and most of them (59.42%) were willing to pay 1000–2000 RMB. Moreover, we discovered that individuals (26.29%) that supported the NBGS were willing to pay more than others (> 2000RMB), whilst those who were not in favour (30.26%) of the NBGS were more likely to keep lower expenses (< 1000RMB) (Table 3).

**Table 3:** Participants’ expectation on undergoing genetic screening

	Is it necessary to undergo genetic screening?			Value	P
	Total (n = 1141, 100%)	Yes (n = 989, 86.68%)	No/I don’t know (n = 152, 13.32%)		
Do you think it is necessary to increase the number of diseases in NBS?					
Yes	930 (81.51%)	861 (87.06%)	69 (45.39%)		
No	15 (1.31%)	11 (1.11%)	4 (2.63%)	154.865	P < 0.05
Have no idea	34 (2.98%)	22 (2.22%)	12 (7.90%)		
Consult doctor	162 (14.20%)	95 (9.61%)	67 (44.08%)		
How many new diseases do you wish to be added to NBS?					
As many and comprehensive as possible	821 (71.96%)	734 (74.22%)	87 (57.24%)	88.685	P < 0.05
Add another 10–50 types with a relatively high incidence	257 (22.52%)	225 (22.75%)	32 (21.05%)		
No need to add any new diseases	63 (5.52%)	30 (3.03%)	33 (21.71%)		
The cost of NBS may increase with the number of screening diseases. How much are you willing to pay for the NBS after expanding the types of diseases?					
< 1000 RMB	181 (15.86%)	135 (13.65%)	46 (30.26%)		
1000–2000 RMB	678 (59.42%)	594 (60.06%)	84 (55.26%)	30.875	P < 0.05
> 2000 RMB	282 (24.72%)	260 (26.29%)	22 (14.48%)		
What is the minimum detection rate for each disease in newborn genetic screening that you can accept?					
> 95%	625 (54.78%)	554 (56.02%)	71 (46.71%)		
> 75%	163 (14.28%)	148 (14.96%)	15 (9.87%)		
> 60%	102 (8.94%%)	87 (8.80%)	15 (9.87%)	15.290	P < 0.05
> 30%	251 (22.00%)	200 (20.22%)	51 (33.55%)		
For low onset genetic diseases, the detection rate of genetic screening is low. What is the minimum detection rate for these diseases in newborn genetic screening that you can accept?					
> 80%	586 (51.36%)	519 (52.48%)	67 (44.08%)		
> 65%	194 (17.00%)	164 (16.58%)	30 (19.74%)	8.576	P < 0.05
> 50%	123 (10.78%)	111 (11.22%)	12 (7.89%)		
> 30%	238 (20.86%)	195 (19.72%)	43 (28.29%)		

### Crowds' analysis of NBGS supporters

Approximately 86.68% of participants agreed that it is essential to conduct the NBGS as a supplement to the traditional NBS to ascertain the genetic causes of diseases and intervene promptly (Table 4). There were no significant variations in gender, age, race, genetic background, or life attitudes between those who favoured the NBGS and those who did not or were uncertain. People who supported the NBGS were generally more worried about diagnosing genetic diseases in children that could not be treated.

**Table 4:** Views of different participants on genetic screening

	Is it necessary to undergo genetic screening?			Value	P
	Total (n = 1141, 100%)	Yes (n = 989, 86.68%)	No/I don’t know (n = 152, 13.32%)		
Gender					
Male	271 (23.75%)	235 (23.76%)	36 (23.68%)	0.00	P > 0.05
Female	870 (76.25%)	754 (76.24%)	116 (76.32%)		
Age (years old)					
≤ 24	41 (3.59%)	37 (3.74%)	4 (2.63%)		
25–29	443 (38.83%)	380 (38.42%)	63 (41.45%)	5.995	P > 0.05
30–34	490 (42.94%)	418 (42.27%)	72 (47.37%)		
≥ 35	167 (14.64%)	154 (15.57%)	13 (8.55%)		
Have you had family history of genetic disease?					
Yes	3 (0.26%)	3 (0.30%)	0 (0.00%)	0.462	P > 0.05
No	1138 (99.74%)	986 (99.70%)	152 (100%)		
Nation					
Han nationality	1117 (97.90%)	969 (97.98%)	148 (97.37%)	0.238	P > 0.05
Others	24 (2.10%)	20 (2.02%)	4 (2.63%)		
Had any abnormalities in your/your spouse's fetus during pregnancy?					
Yes	34 (2.98%)	32 (3.24%)	2 (1.32%)	1.680	P > 0.05
No	1107 (97.02%)	957 (96.76%)	150 (98.68%)		
If your child is diagnosed with an inherited genetic disease but not treated, will you worry about the aggravation of your child's condition?					
Yes	1120 (98.16%)	977 (98.79%)	143 (94.08%)	16.162	P < 0.05
No	21 (1.84%)	12 (1.21%)	9 (5.92%)		
Educational background					
Below high school	53 (4.64%)	38 (3.84%)	15 (9.87%)	23.727	P < 0.05
High school and technical secondary school	103 (9.03%)	83 (8.39%)	20 (13.16%)		
Junior college/Vocational College	242 (21.21%)	200 (20.22%)	42 (27.63%)		
Undergraduate or above	743 (65.12%)	668 (67.55%)	75 (49.34%)		
Family income					
< 100 thousand RMB	187 (16.39%)	137 (13.85%)	50 (32.89%)		
100–250 thousand RMB	577 (50.57%)	508 (51.37%)	69 (45.39%)	40.277	P < 0.05
260–400 thousand RMB	255 (22.35%)	227 (22.95%)	28 (18.42%)		
> 400 thousand RMB	122 (10.69%)	117 (11.83%)	5 (3.30%)		
If your child is diagnosed with an inherited genetic disease and requires long-term (lifelong) treatment, how do you think it will affect your family life status?					
Decrease the quality of life, increase daily burden and anxiety, and life becomes negative	267 (23.40%)	227 (22.95%)	40 (26.32%)	0.831	P > 0.05
Grateful for early detection and treatment for the disease which improves the child’s quality of life	874 (76.60%)	762 (77.05%)	112 (73.68%)		

**Fig. 2:**
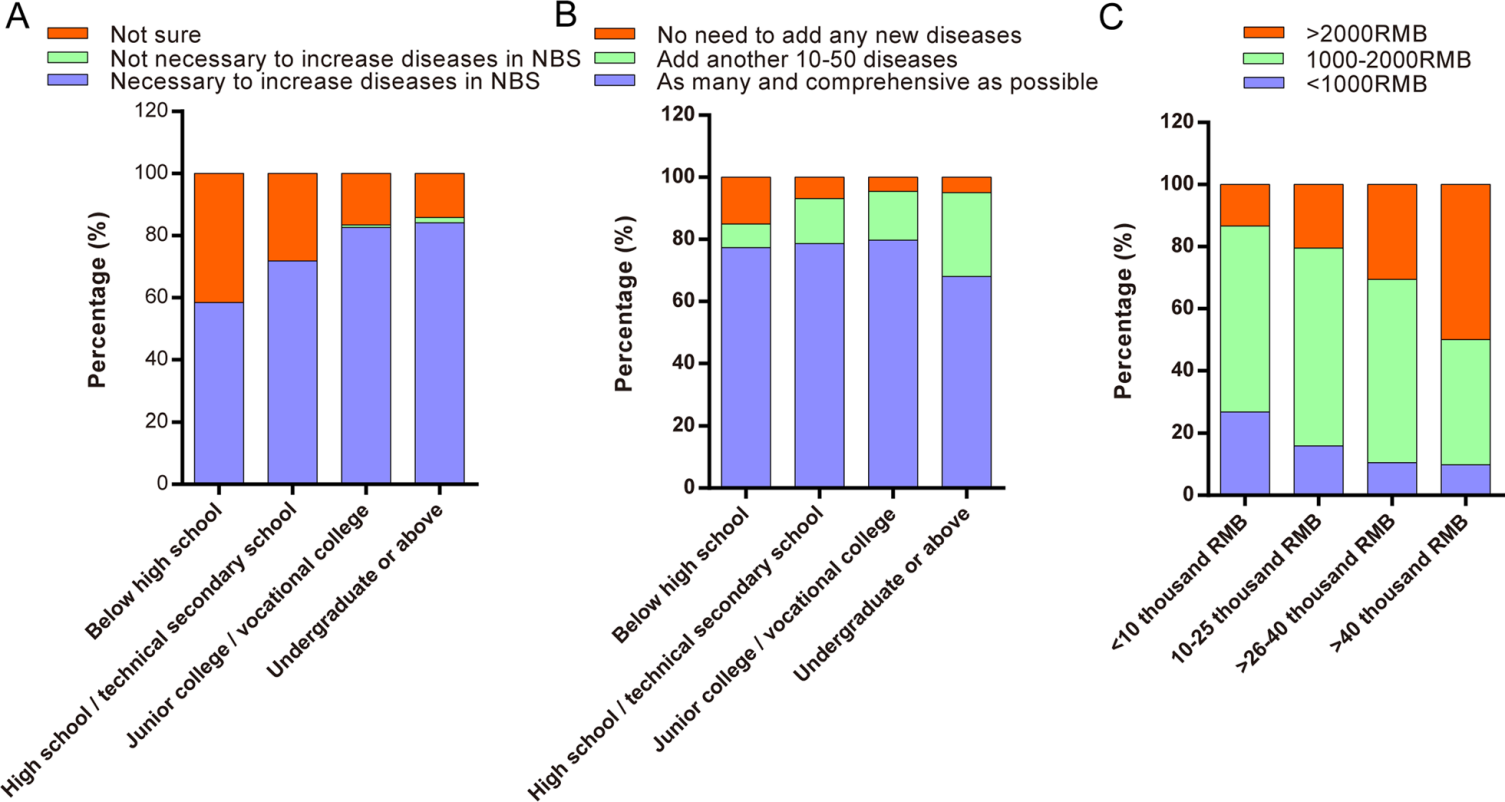
Cross-over analysis of the participants. **A** The participants had different education degrees and they differed in opinion regarding whether it was necessary to increase the number of diseases in NBS (Q12). **B** The views of the participants with different education degrees on how many new diseases were added to NBS (Q13). **C** The views of the participants of different family incomes concerning how much they would be willing to pay for the NBS after expanding the types of diseases (Q14)

Significant differences were observed in terms of education level and family income. Among the population that supported the NBGS, a highly-educated population accounted for the substantial part (67.55% vs. 49.34%), while among the non-supportive population, the less-educated population was relatively significant. In addition, it was also found that people with higher education levels had a more explicit attitude towards the proposal of increasing the number of diseases detected by NBS (Fig. 2A). Further, some participants with higher education levels possessed a conservative view about the possible increase in diseases detected in NBS, believing that a moderate increase of 10–50 types was enough (Fig. 2B). Besides, people with low education degrees were more uncertain and pessimistic about adding diseases to the NBS project.

The current study also revealed that individuals who supported the NBGS had a significant proportion of high-income participants (> 400,000 RMB, 11.83% vs. 3.3%) (Table 4). Furthermore, high-income families were more willing than low-income families to tolerate increasing testing costs (Fig. 2C).

### Consideration and expectations for NBGS

Regarding the implementation of the NBGS project, participants were more willing to learn about it through face-to-face consultation with doctors (91.76%), followed by brochures and educational videos (59.86%), small lessons for pregnant women (54.51%), and official hospital accounts (50.04%), but seldom choose to search and inquire online by themselves (27.96%). The participants were willing to actively cooperate during the diagnostic processes when the initial screening results were positive (98.51%), and the people who favoured the NBGS were more active (99.70% vs. 90.79%). The participants (98.07%) also desired to know when the suspected pathogenic genes were not related to the clinical manifestations. They (93.69%) were also willing to inform their children about their NBGS results when they became adults and advised them to undertake further carrier screening with their partners before starting a family (Table 5).

**Table 5:** Participants’ demands after undergoing the genetic screening

	Is it necessary to undergo genetic screening?			Value	P
	Total (n = 1141, 100%)	Yes (n = 989, 86.68%)	No/I don’t know (n = 152, 13.32%)		
Which ways would you like to learn more about NBGS? (Multiple options)					
Consult doctors face to face	1047 (91.76%)	917 (92.72%)	130 (85.53%)		
Brochure or education video of outpatient waiting area	683 (59.86%)	599 (60.57%)	84 (55.26%)		
Get online and research by oneself	319 (27.96%)	274 (27.70%)	45 (29.61%)	1.781	P > 0.05
Study or lecture for pregnant women	622 (54.51%)	549 (55.51%)	73 (48.03%)		
The official account of hospital	571 (50.04%)	507 (51.26%)	64 (42.11%)		
Will you actively obtain a genetic diagnosis after a positive genetic screening result?					
Yes, actively carry out a genetic diagnosis to clarify the disease	1124 (98.51%)	986 (99.70%)	138 (90.79%)	71.218	P < 0.05
No need, genetic screening is equal to genetic diagnosis	17 (1.49%)	3 (0.30%)	14 (9.21%)		
If your child's genetic screening results reveal other suspected pathogenic genes, but are not related to the clinical phenotype, would you like to be informed?					
Yes, I need to know	1119 (98.07%)	978 (98.89%)	141 (92.76%)	26.135	P < 0.05
Don’t want to know, to reduce the anxiety after knowing the result	22 (1.93%)	11 (1.11%)	11 (7.24%)		
If you know that your children have carried pathogenic genes through genetic screening, will you tell them when they become adults?					
Yes, and recommend them to do a carrier screen with their partner before giving birth to their child	1069 (93.69%)	935 (94.54%)	134 (88.16%)	9.077	P < 0.05
Will not tell them, to reduce their anxiety after knowing the results	72 (6.31%)	54 (5.46%)	18 (11.84%)		

As society and science have progressed, people have become more aware and dissatisfied with the existing NBS program. Especially with the NBGS, which may supplement newborn screening and allow them to understand more about congenital diseases.

## Discussion

Based on the present study, it was found that the factors that can potentially influence the support of the people for NBGS were mainly related to personal cognition, education, and family income. Among those who did not support NBGS, 49.34% were highly educated, while 23.03% belonged to the low-educated group. Of the participants supporting NBGS, people with high education credentials accounted for 67.55%, whereas those with low education profiles accounted for only 12.23%. Similarly, in the non-supportive NBGS population, 3.3% had an annual family income of more than 400,000 RMB, and 32.89% had an annual family income of less than 100,000 RMB. Participants with a yearly household income of less than 100,000 RMB accounted for only 13.85% of those who supported NBGS. In contrast, those with an annual household income of more than 400,000 RMB accounted for only 11.83% (Table 4).

Our findings also revealed that for the future clinical development of NBGS, it is critical to do an exceptional job sharing relevant NBGS knowledge. The general public must fully comprehend the importance of NBGS development. We observed that participants preferred to learn passively rather than actively to understand NBGS content (Table 5).

China has unveiled a new policy of three-child per family, which has recommended that a couple can have up to three children. Thus, the importance of the NBGS project is not only to efficiently screen out newborn diseases on time but also to combine it with genetic counselling to guide parents in reproducing, which is crucial in producing healthy children. In addition, we also found that the majority (15.57% vs. 8.55%) of the adults (≥ 35 years old) were willing to support the development of NBGS, which makes us gratified. The benefit of the three-child policy is that the number of advanced maternal age will inevitably increase, while the pregnancy outcomes in older couples have always been a cause for concern [20, 21]. After the project is successfully implemented, older people's support for NBGS will facilitate genetic counselling for advanced maternal age couples.

The actual development of NBGS still needs to go through a relatively long process of exploration, including the specific inclusion of conditions and schemes for the NBGS. Most people believe that more diseases should be included regarding the population's views on the number of diseases screened by NBGS. A small number of people indicated that the suitable amount is enough; this part of the population was also dominated by highly-educated people (Fig. 2), thereby showing a more conservative consideration for supporting the increase in the number of NBGS diseases. The number of diseases that can be screened is limited. As a result, it is crucial to analyze all aspects of the need for early screening, such as the severity of the disease and whether or not it can be treated, because early treatment can improve the prognosis and minimize related morbidity [11, 22, 23]. A strategy for early intervention could potentially generate accurate predictions for disorders affecting infants to children that pose substantial risks but are treatable. Thus, such diseases can be considered the most suitable for the NBGS project inclusion, which will increase the family's program acceptance rate. Simultaneously, care should be taken to avoid including disorders with unknown genotype–phenotype connections, mild phenotypes that may not require intervention, or diseases with late-onset, to avoid the development of "patients in waiting" and the negative effects of overmedicalization [24]. Overmedicalization in this context refers to interventions that are not medically required or evidence-based, which may raise costs and cause concern in these families. The choice of detection technology requires consideration of its cost-effectiveness. In the case of fully considering the national conditions, the detection technology with low cost, simple operation, and short reporting period would be selected [25].

Since the phenotype is not only associated with genotype but also has distinct phenotypic variability, entirely replacing traditional biochemical screening with genetic testing may not be an appropriate screening optimization technique. The organic combination of NBGS and standard biochemical screening may have unexpected effects. The implementation of NBGS requires careful consideration of the above aspects and a comparative analysis of cost-effectiveness before arriving at the best implementation plan that the families ultimately require. In addition, the development of NBGS should also pay attention to ethical issues, especially for genetic counselling, psychological counselling, and carrier information feedback for screen-positive family members. Previous studies have suggested both benefits and risks in feeding information about newborn carriers to newborn families [26, 27]. Nonetheless, we must conduct more appropriate and targeted assessments for different groups and modify the report content based on the evaluation results.

NBGS is a novel concept that has recently been introduced, and it can be considered a significant generation milestone in the field of newborn screening. With the emergence of next-generation sequencing technology, the detection of various inherited diseases is no longer limited to biochemical methods. Thus, analyzing the various diseases from the genetic perspective could help assist in intervention, diagnosis, and treatment. For the best choice of NBGS methods, it is still important to compare and evaluate the cost, detection cycle, DNA coverage, ease of operation, and the population they can be used on. The clinical development of NBGS is also an inevitable trend, but it still needs continuous improvement in the advancement process for everyday use.

We understood the attitudes and beliefs of the reproductive-aged population towards NBGS and found that most individuals were willing to accept NBGS programs. Notably, those with a high level of education and income provide a solid foundation for the future development of NBGS. However, this study also has limitations. The number of male participants was limited, making it difficult to effectively assess the cognitive differences between males and females on many issues.

When "global health" and "social justice" have taken on particular relevance due to the COVID 19 pandemic and vaccine processes, the development of NBGS seems more controversial. In light of current findings, people are motivated to improve the existing NBS on the one hand because they foresee future improvements in their quality of life. The cost of NBGS testing, on the other hand, may hinder the development of NBGS in countries with low-income and lower literacy rates. Yet, the development of NBGS may be reasonably simple and easy for high-income countries and for educated people who understand its importance for the family in the longer term. However, if we want to carry out NBGS in low- and middle-income countries, the income level of low- and middle-income people still needs to be mainly considered, which comprises most of the population. As a middle-income country with a large population, China should consider three leading solutions: (i) improving testing technology to reduce testing costs, (ii) improving the part of medical insurance for NBS, and (iii) giving appropriate government subsidies until the program has been highly accepted and appreciated by the community. The experience and results obtained in implementing and developing NBGS in China may provide reference and help develop NBGS in other developing countries. Prenatal and postnatal care should be paid more attention to the long-term development of human beings. The smooth development of NBGS can improve the screening efficiency of newborn genetic diseases, early detection, and early treatment, providing fertility guidance for the family of the patient children and enhancing the fertility rate. The experience of China's NBGS can also be used for research in other countries to promote global health as a model study.

## Supplementary Information

**Additional file 1. Table S1**: Questionnaire designed.

## Data Availability

The datasets used and/or analyzed during the current study are available from the corresponding author on reasonable request.
