# Supplementary material for: Current attitudes and preconceptions on newborn genetic screening in the Chinese reproductive-aged population
Source: Orphanet J Rare Dis. 2022 Aug 26;17:322. doi: 10.1186/s13023-022-02474-8 (PMC9412816; doi:10.1186/s13023-022-02474-8)
Supplement: Supplementary file 1 — Additional file 1. Table S1: Questionnaire designed. [file 13023_2022_2474_MOESM1_ESM.pdf]

Table S1. Questionnaire designed.

No.	Question
Q1	Age
	18-24 years old
	25-32 years old
	>33 years old
Q2	Family history of genetic disease
Q3	Educational background (bachelor or above)
Q4	Family income:
	<10 thousand RMB
	10-25 thousand RMB
	26-40 thousand RMB
	>40 thousand RMB
Q5	Does the participant know about the NBS[a] program?
Q6	Ways to obtain NBS propaganda and education:
	A detailed explanation by a doctor or nurse
	Self-study through propaganda materials or WeChat mini programs
	Other ways
Q7	Do you think if both couples do not have genetic diseases, their child will not have a genetic disease?
	Yes.
	No.
	I don't know.
Q8	If the NBS results are negative, does it mean that the baby will not have an IMD[b]?
	Yes.
	No.
	I don't know.
Q9	Do you know what it means to be suspiciously positive for NBS?
	It means that there have abnormal indicators and needs to be further re-examined.
	It means that a disease has been diagnosed.
	I don't know, it’s probably meaningless.
Q10	If your child is diagnosed with an IMD and requires long-term (lifelong) treatment, how do you think it will affect your family life status?

	Decrease the quality of life, increase daily burden and anxiety, and life becomes negative<br>Grateful for early detection and treatment for the disease which improves the child’s quality of life.
Q11	Clear about what diseases are included in the current NBS in Nanjing. (29 kinds of inherited metabolic diseases, hearing impairment and congenital heart disease)
Q12	Believed it is necessary to increase the number of diseases in NBS (such as deafness, severe combined immunodeficiency disease, progressive muscular dystrophy, and lysosomal disease, etc.).
Q13	How many new diseases do you wish to be added to NBS?<br>As many and comprehensive as possible<br>Add another 10-50 types with a relatively high incidence<br>No need to add any new diseases
Q14	The cost of NBS may increase with the number of screening diseases. How much are you willing to pay for the NBS after expanding the types of diseases? (Self-paid part, non-medical insurance reimbursement category)<br><1000 RMB<br>1000-2000 RMB<br>>2000 RMB
Q15	Consider it is necessary to do genetic screening for genetic diseases of newborns, to find out the genetic causes as early as possible, intervene and treat them in time.
Q16	What is the minimum detection rate for each disease in newborn genetic screening that you can accept?<br>>95%<br>>75%<br>>60%<br>>30%
Q17	For low onset genetic diseases, the detection rate of genetic screening is low. What is the minimum detection rate for these diseases in newborn genetic screening that you can accept?<br>>80%<br>>65%<br>>50%<br>>30%
Q18	Will you actively obtain a genetic diagnosis after a positive genetic screening result?<br>Yes, actively carry out a genetic diagnosis to clarify the disease.

	Not necessary, genetic screening is equal to genetic diagnosis.
Q19	If your child's genetic screening results reveal other suspected pathogenic genes, but are not related to the clinical phenotype, would you like to be informed?
	Yes, I need to know
	Don't want to know, to reduce the anxiety after knowing the result
Q20	If you know that your children have carried pathogenic genes through genetic screening, will you tell them when they become adults?
	Yes, and recommend them to do a carrier screen with their partner before giving birth to their child.
	Will not tell them, to reduce their anxiety after knowing the results.

[a], NBS, newborn screening; [b], IMD, inherited metabolic diseases.
